# Can radiology requisition quality reflect clinical reasoning? Insights from a RI-RADS evaluation of emergency CT referrals

**DOI:** 10.1186/s13244-025-02111-5

**Published:** 2025-10-16

**Authors:** Xianwei Liu, Zhengguang Xiao, Qinghua Min, Tianhao Wu, Yue Xing, Yangfan Hu, Defang Ding, Shun Dai, Junjie Lu, Jiarui Yang, Yue Li, Yang Song, Minda Lu, Jingshen Chu, Huan Zhang, Weiwu Yao, Jingyu Zhong

**Affiliations:** 1 Department of Imaging, Tongren Hospital, Shanghai Jiao Tong University School of Medicine, Shanghai, China; 2 Shanghai Key Laboratory of Flexible Medical Robotics, Tongren Hospital, Institute of Medical Robotics, Shanghai Jiao Tong University, Shanghai, China; 3 Department of Epidemiology and Population Health, Stanford University School of Medicine, Stanford, California, CA USA; 4 Department of Biomedical Engineering, Boston University, Boston, MA USA; 5 Jacobi Medical Center, Albert Einstein College of Medicine, New York, NY USA; 6 MR Research Collaboration Team, Siemens Healthineers Ltd., Shanghai, China; 7 MR Application, Siemens Healthineers Ltd., Shanghai, China; 8 Editorial Office of Journal of Diagnostics Concepts & Practice, Department of Science and Technology Development, Ruijin Hospital, Shanghai Jiao Tong University School of Medicine, Shanghai, China; 9 Department of Radiology, Ruijin Hospital, Shanghai Jiao Tong University School of Medicine, Shanghai, China; 10 College of Health Science and Technology, Shanghai Jiao Tong University School of Medicine, Shanghai, China; 11 Shanghai Key Laboratory of Gastric Neoplasms, Department of Surgery, Shanghai Institute of Digestive Surgery, Ruijin Hospital, Shanghai Jiao Tong University School of Medicine, Shanghai, China

**Keywords:** Multidetector computed tomography, Emergency medicine, Practice guidelines, Referral and consultation, Quality improvement

## Abstract

**Objectives:**

To determine radiology requisition quality using reason for exam imaging reporting and data system (RI-RADS), and the associated clinical variables, and whether it can reflect the clinical reasoning quality in emergency CT referrals.

**Materials and methods:**

This single-center retrospective study randomly selected emergency CT referrals between 01 January 2024 and 31 December 2024. One radiologist scored the requisition quality using the RI-RADS, and assessed the clinical reasoning quality, evaluating the extent to which the differential diagnoses on the requisition form matched the CT diagnosis on the issued report. The clinical variables associated with RI-RADS A were investigated using logistic regression analysis. The clinical reasoning quality among different RI-RADS grades was compared.

**Results:**

We included 1291 emergency CT referrals. RI-RADS grades A (adequate), B (barely adequate), C (considerably limited), D (deficient), and X (no information) were assigned to 287 (22.2%), 71 (5.5%), 851 (65.9%), 53 (4.1%), and 29 (2.2%) requisitions, respectively. The requisitions from the fever clinic and thoracic surgery were less likely to be assigned to RI-RADS A compared to internal medicine (odds ratio 0.10–0.11), and so were those of abdomen and head scans compared to chest scans (odds ratio 0.06–0.30). The RI-RADS A requisitions had better clinical reasoning quality than those of RI-RADS grades C, D, and X (all adjusted *p* < 0.001), but not those of RI-RADS B (adjusted *p* = 0.100).

**Conclusion:**

The majority of emergency CT requisitions were inadequate, especially those from the fever clinic and thoracic surgery. Suboptimal requisitions were associated with poorer clinical reasoning quality.

**Critical relevance statement:**

Radiologists’ concerns are valid that low-quality radiology requisitions may reflect poorer clinical reasoning. The use of standardized requisition frameworks like RI-RADS may help bridge the gap between diagnostic imaging and clinical reasoning, promoting safer and more effective patient care.

**Key Points:**

- Radiologists often complain about low-quality radiology requisitions with low clinical reasoning quality from clinicians.
- The radiological requisitions with lower quality according to RI-RADS were associated with worse clinical reasoning quality.
- Improving the quality of radiological requisitions after detailed clinical reasoning is warranted to improve radiology workflow for better medical practice.

**Graphical Abstract:**

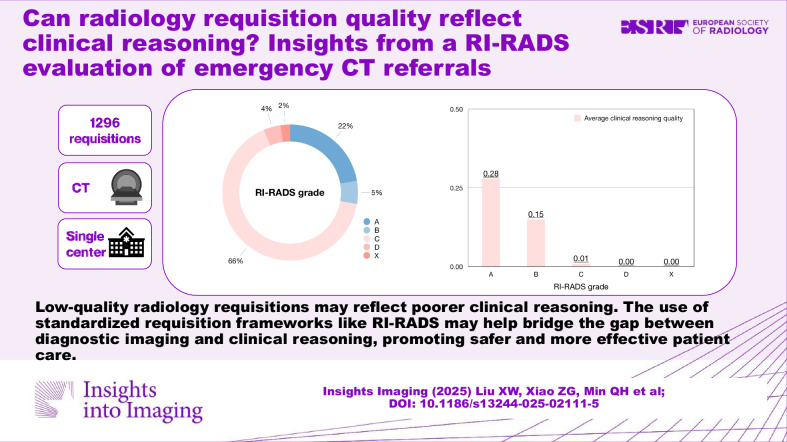

## Introduction

The volume of radiological examinations is continuously increasing worldwide [1, 2], especially those of emergency examinations [3, 4]. With the application of picture archiving and communication systems (PACS), radiologists can assess images instantly and promote efficient patient care to respond to the increasing workload [5–7], whereas face-to-face communication among radiologists, clinicians, and patients is reduced [8, 9]. Radiologists now rely more on the requisitions from clinicians to be informed about the patients [10]. Adequate clinical information can improve the protocol selection as well as the interpretation accuracy [11–16]. However, clinicians often fail to provide sufficient clinical information for radiologists, and radiologists usually cannot take enough time to extract relevant clinical information for interpreting the images from the vast amount of data in the electronic medical records [8, 9].

The standardization of requisitions is expected to solve the problem [17–19]. An Italian expert panel has developed a structured requisition form to guide clinicians to provide sufficient clinical information in musculoskeletal imaging [20]. Another expert panel from Australia listed items for information medically pertinent in acute CT requisitions to provide criteria standards for adequate clinical information in emergency CT requisitions [21]. The reason for exam imaging reporting and data system (RI-RADS) is a tool for quality assessment of all radiological examination requisitions concerning three key elements of impression, clinical information, and diagnostic question, to categorize the requisitions into a five-point scale [22, 23]. The tool can be used regardless of the subspecialty or imaging modality, and may improve the quality of patient care and reduce lawsuits against radiologists [23]. The RI-RADS has been demonstrated to have substantial reliability and ability to identify inadequate radiological examination requisitions [24–26]. Meanwhile, the referring clinicians became worse at clinical reasoning over the years [27]. However, it is unclear whether RI-RADS can reflect the clinical reasoning quality of the referring clinicians. Accordingly, we hypothesized that the key elements of RI-RADS are associated with the clinical reasoning quality. Here, we investigated their association in emergency CT referrals, to emphasize the necessity of improving requisitions from both perspectives of the demands of radiologists and a culture of quality improvement.

Therefore, our study aimed to determine radiology requisition quality using RI-RADS and associated clinical variables, and whether it can reflect the clinical reasoning quality in emergency CT referrals.

## Materials and methods

### Study design and sample size

Our institutional ethics approval committee has approved this retrospective study (K2024-083-01), and the written informed consent was waived. We calculated the required sample size according to the formula, $N=\tfrac{{Z}_{\alpha /2}^{2}p(1-p)}{{\delta }^{2}}$, where *N* is the sample size, *p* is the proportion, and *δ* is the estimation error. The expected proportion of RI-RADS grade A was considered as 0.236 according to a previous study [28]. We set the α error probability and estimation error to 0.05 and 10% of the expected proportion of 0.0236. The required sample size resulted in 1244 requisitions. As our institution has 89,524 emergency CT requisitions in the year of 2024, we decided to randomly screen 1368 requisitions for eligibility assessment, considering a drop rate of 10%. The requisitions were selected using a random number generator (https://www.random.org).

### Requisition selection

Our institution is a 1200-bed tertiary university hospital with an outpatient volume of 2.3 million and an inpatient volume of 70 thousand. All the emergency CT requisitions originated from all departments, including the emergency department. All the requisitions in the study were neither evaluated nor discussed in advance, as the huge amount of patient volume and rush workflow did not allow radiologists to discuss the requisitions of CT scans case by case. In our institution, clinicians can request for emergency CT scans at will, resulting in more than 89,000 emergency CT requisitions in the year of 2024. All scans should be performed within half an hour, and all the initial radiological reports should be written within half an hour after the scan. All the initial radiological reports would be later issued by a second subspecialty fellowship-trained radiologist within 24 h after the scan. All the requisitions were screened for eligibility by two radiologists with 6 and 10 years of experience in CT image interpretation (J.Z. and X.L.), respectively. The inclusion criteria for the requisitions were (a) CT requisitions marked as emergency; (b) requisitions between 01 January 2024 and 31 December 2024. The exclusion criteria for the requisitions were (a) requisition form not available; (b) missing CT image or radiological report; (c) incomplete clinical data. Discrepancies were resolved by discussion or consulting a third radiologist with 22 years of experience (Z.X.).

### Data extraction

The same two radiologists (J.Z. and X.L.) extracted all the data. For each CT requisition, the following clinical variables were extracted: age, gender, requesting specialty (chest pain center, emergency medicine, fever clinic, general surgery, gynecology and obstetrics, internal medicine, neurology, neurosurgery, orthopedics, otolaryngology, thoracic surgery, or others), body region (abdomen, cardiovascular, chest, extremity, head, neck, spine, or multiple body regions), time of examination (office hours, 8:00–17:00, before midnight, 17:00–24:00, or after midnight, 0:00–8:00), and use of intravenous CT contrast agents (yes or no). Discrepancies were resolved by discussion or consulting a third radiologist with 22 years of experience (Z.X.).

### RI-RADS grade evaluation

A radiologist with 10 years of experience in CT image interpretation (X.L.) reviewed all the emergency CT requisitions and assigned an RI-RADS grade to each requisition. The assessment was only based on the requisition form that was in an instructed format, which was digitally available for radiologists when reading CT images and writing reports. The additional information from other electronic medical records, or notes that were taken by radiologists via phone calls or discussions with clinicians, was excluded. The RI-RADS evaluates the requisition quality according to three key categories of impression (yes, or no), clinical information (yes, barely yes, or no), and diagnostic question (yes, or no) (Table 1) [22, 23]. The RI-RADS has five grades, namely RI-RADS A (adequate), RI-RADS B (barely adequate), RI-RADS C (considerably limited), RI-RADS D (deficient), and RI-RADS X (no information) (Table 1) [22, 23]. A second radiologist with 22 years of experience in CT image interpretation (Z.X.) independently rated the RI-RADS grades of a random subsample of 100 requisitions that were assigned by the first radiologist, to determine inter-rater agreement. Discrepancies were resolved by discussion or consulting a third radiologist with 24 years of experience (Q.M.).

**Table 1:** RI-RADS key categories and RI-RADS grading

	Description	Example
Key category		
Impression	Working or differential diagnosis	Yes: “Acute abdomen”, “Suspect for pancreatitis”, “Shoulder dislocation” No: “Health checkup”, “Request for examination”, “Not specific disease of urinary system”, “11111”, “/”
Clinical information	Signs and symptoms, chronicity of current episode, location of signs and symptoms, pertinent past medical/surgical history, pertinent laboratory findings, and previous imaging reports when available	Yes: “Upper abdominal pain after dinner of greasy food for 6 h. Elevated serum amylase. History of fatty liver, hyperlipidemia. No diabetes.” Barely yes: “Severe back pain for 1 h. History of nephrolith.” No: “Request for examination”, “Normal”, “11111”, “/”
Diagnostic question	Confirmation/exclusion of diagnosis, grading/staging, pre-operative planning, follow-up of progress, or response to treatment	Yes: “Rule out foreign body in stomach”, “Response to antibiotics in a pneumonia patient”, “Confirmation of bowel obstruction” No: “Pain”, “11111”, “/”, “Further evaluation”
RI-RADS grading		
Grade A	Adequate: all key categories of information included	Abdomen CT: “Acute abdomen. Abdominal pain for 2 days. No flatus or defecation. History of right colon cancer. Colectomy 2 years ago. Liver metastasis. History of bowel obstruction 3 months ago. Confirmation of bowel obstruction.”
Grade B	Barely adequate: all key categories of information included, some clinical findings missing	Chest CT: “Pneumonia. Confirmed pneumonia in another hospital. Slightly elevated CRP, WBC. Evaluation of pneumonia severity.”
Grade C	Considerably limited: two categories of information included	Head CT: “Head injury. No diabetes. No hypertension. Exclusion of intracerebral hemorrhage.”
Grade D	Deficient: one category of information was included	Head CT: “History of cerebral Infarction” Abdomen CT: “Abdominal pain”
Grade X	Deficient: no category of information included	Chest CT: “Come by ambulance” Knee CT: “11111”

### Clinical reasoning assessment

A radiologist with 10 years of experience in CT image interpretation (X.L.) evaluated the clinical reasoning quality [27]. The clinical reasoning quality was quantified by comparing the differential diagnosis made by the referring clinicians on the requisition form, and the final CT diagnosis on the double-read reports issued by subspecialty fellowship-trained radiologists. If no differential diagnosis was considered by the clinician, or none of the differential diagnoses on the requisition form was matched to the final CT diagnosis on the report, 0 points were assigned. Each differential diagnosis on the requisition form that matched the final CT diagnosis on the report received 1 point. The clinical reasoning score was calculated by dividing the number of matched differential diagnoses by the total number proposed on the requisition form. The range of clinical reasoning quality is 0 to 1. The larger the number, indicates better the clinical reasoning quality. For example, if the clinical differential diagnosis included bowel obstruction, urolithiasis, cholecystitis, and pancreatitis, and the CT examination demonstrated pancreatitis, the clinical reasoning quality would be 1/4 = 0.25.

### Data analysis

The statistical analysis was conducted using the R language (version 4.2.1; https://www.r-project.org/) within RStudio (version 1.3.1093; https://posit.co/) by a radiologist with 6 years of experience in radiology research (J.Z.) under the supervision of a biostatistician (J.L.). The two-tailed alpha level was set at 0.05, unless otherwise specified. The inter-rater agreement of RI-RADS categories and RI-RADS grades was assessed by absolute agreement. The clinical variables were evaluated to determine whether they were associated with RI-RADS A. They were first tested using univariate logistic regression to determine whether they were associated with RI-RADS A, with an alpha level of 0.10. The clinical variables that were considered as associated with RI-RADS A grade were further assessed using multiple logistic regression analysis, to estimate the adjusted odds ratio (OR) and 95% confidence interval (CI) of whether factors were associated with RI-RADS A, using an alpha level of 0.05. A similar analysis was also performed for the RI-RADS A, or B grade (adequate) vs RI-RADS C, D, or X grade (inadequate) requisitions. The difference among RI-RADS grades was evaluated using Mann–Whitney *U*-test or the Kruskal–Wallis *H*-test for continuous variables, and the Chi-square test, or Fisher's exact test for categorical variables. When a significant difference was found, *post hoc* pairwise comparisons were conducted with Bonferroni correction.

## Results

### Characteristics of requisitions

**Fig. 1:**
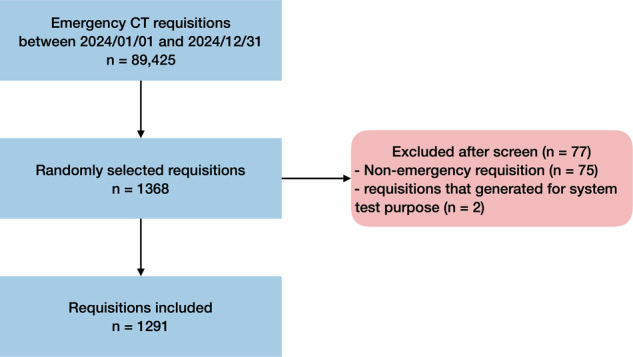
Flowchart of requisition inclusion. There were 89,425 emergency CT requisitions from 01 January 2024 and 31 December 2024 in our institution, in which 1368 requisitions were randomly selected and screened for eligibility assessment. After exclusion of 75 non-emergency requisitions and 2 requisitions that were generated for system test purposes, respectively, 1291 requisitions were included for RI-RADS evaluation

Our study included 1291 requisitions for evaluation after excluding 75 non-emergency requisitions and 2 requisitions that were generated for system test purposes (Fig. 1). The requisitions were for patients with an average ± standard deviation, median (interquartile range) age of 54.0 ± 21.2, 56.0 (34.0) years, and slightly more than half were female (675 requisitions, 52.3%) (Table 2). The distribution of requesting specialty (*p* < 0.001), body region (*p* < 0.001), requisition time (*p* = 0.003), and contrast agent usage (*p* < 0.001) were significantly different among RI-RADS grades.

**Table 2:** Characteristics of included requisitions

Variables	Overall (*n* = 1291)	RI-RADS					*p* value
		Grade A (*n* = 287)	Grade B (*n* = 71)	Grade C (*n* = 851)	Grade D (*n* = 53)	Grade X (*n* = 29)	
Age, year, mean ± standard deviation, median (interquartile range)	54.0 ± 21.2, 56.0 (34.0)	53.3 ± 22.3, 57.0 (37.5)	56.8 ± 18.6, 62.0 (28.5)	54.1 ± 21.1, 56.0 (33.0)	57.5 ± 20.2, 61.0 (29.0)	45.2 ± 18.8, 37.0 (29.0)	0.077
Gender, *n* (%)							0.129
Male	616 (47.7)	139 (48.4)	25 (35.2)	407 (47.8)	27 (50.9)	18 (62.1)	
Female	675 (52.3)	148 (51.6)	46 (64.8)	444 (52.2)	26 (49.1)	11 (37.9)	
Requesting specialty, *n* (%)							< 0.001
Chest pain center	36 (2.8)	9 (3.1)	1 (1.4)	26 (3.1)	0 (0.0)	0 (0.0)	
Emergency medicine	108 (8.4)	29 (10.1)	2 (2.8)	74 (8.7)	2 (3.8)	1 (3.5)	
Fever clinic	161 (12.5)	12 (4.2)	31 (43.7)	92 (10.8)	5 (94)	21 (72.4)	
General surgery	159 (12.3)	23 (8.0)	3 (4.2)	125 (14.7)	7 (13.2)	1 (3.5)	
Gynecology and obstetrics	15 (1.2)	3 (1.1)	0 (0.0)	12 (1.4)	0 (0.0)	0 (0.0)	
Internal medicine	354 (27.4)	129 (45.0)	20 (28.2)	197 (23.2)	5 (9.4)	3 (10.3)	
Neurology	206 (16.0)	50 (17.4)	10 (14.1)	144 (16.9)	2 (3.8)	0 (0.0)	
Neurosurgery	81 (6.3)	8 (2.8)	1 (1.4)	65 (7.6)	6 (11.3)	1 (3.5)	
Orthopedics	97 (7.5)	2 (0.7)	3 ().4.2	69 (8.1)	22 (41.5)	1 (3.5)	
Otolaryngology	20 (1.6)	13 (4.5)	0 (0.0)	6 (0.7)	0 (0.0)	1 (3.5)	
Thoracic surgery	36 (2.8)	3 (1.1)	0 (0.0)	30 (3.5)	3 (5.7)	0 (0.0)	
Others	18 (1.4)	6 (2.1)	0 (0.0)	11 (1.3)	1 (1.9)	0 (0.0)	
Body region, *n* (%)							< 0.001
Abdomen	316 (24.5)	53 (18.5)	7 (9.9)	241 (28.3)	12 (22.6)	3 (10.3)	
Cardiovascular	55 (4.3)	29 (10.1)	0 (0.0)	24 (2.8)	2 (3.8)	0 (0.0)	
Chest	440 (34.1)	133 (46.3)	49 (69.0)	228 (26.8)	8 (15.1)	22 (75.9)	
Extremity	49 (3.8)	2 (0.7)	1 (1.4)	33 (3.9)	13 (24.5)	0 (0.0)	
Head	278 (21.5)	34 (11.8)	10 (14.1)	226 (26.6)	6 (11.3)	2 (6.9)	
Multiple body regions	110 (8.5)	24 (8.4)	2 (2.8)	78 (9.2)	5 (9.4)	1 (3.4)	
Neck	13 (1.0)	12 (4.2)	0 (0.0)	1 (0.1)	0 (0.0)	0 (0.0)	
Spine	30 (2.3)	0 (0.0)	2 (2.8)	20 (2.4)	7 (13.2)	1 (3.4)	
Requisition time, *n* (%)							0.003
Office hours	614 (47.6)	144 (50.2)	43 (60.6)	389 (45.7)	31 (58.5)	7 (24.1)	
Before midnight	506 (39.2)	112 (39.0)	21 (29.6)	334 (39.2)	19 (35.8)	20 (69.0)	
After midnight	171 (13.3)	31 (10.8)	7 (9.9)	128 (15.0)	3 (5.7)	2 (6.9)	
Contrast agent usage, *n* (%)							< 0.001
With	70 (5.4)	36 (12.5)	0 (0.0)	32 (3.8)	2 (3.8)	0 (0.0)	
Without	1221 (94.6)	251 (87.5)	71 (100.0)	819 (96.2)	51 (96.2)	29 (100.0)	

### RI-RADS grade of requisitions

**Fig. 2:**
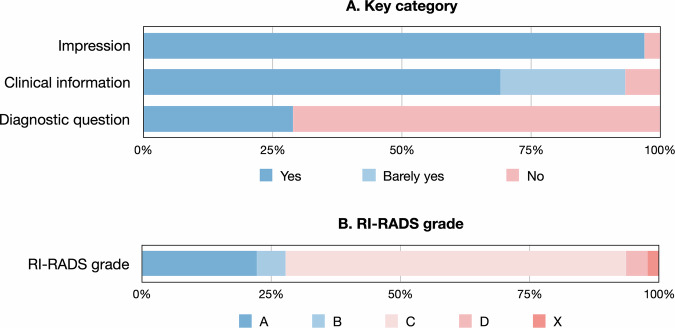
Distribution of RI-RADS key categories and grade. (**A**) RI-RADS key categories, and (**B**) RI-RADS grades of included requisitions were presented using bar plots

The requisitions usually provide impression (1252 requisitions, 97.0% for yes) and clinical information (891 requisitions, 69.0% for yes; 312 requisitions, 16.5% for barely yes), but seldomly diagnostic question (374 requisitions, 29.0% for yes) (Fig. 2). The corresponding requisition quality was poor with only 287 requisitions (22.2%) of RI-RADS A, followed with RI-RADS grades of B (71 requisitions, 5.5%), C (851 requisitions, 65.9%), D (53 requisitions, 4.1%), and X (29 requisitions, 2.2%). The absolute agreements for RI-RADS categories of impression, clinical information, and diagnostic question were 97.0%, 56.0%, and 88.0%, respectively. The absolute agreement for RI-RADS grades was 79.0%.

### Association between RI-RADS grade and clinical variables

A total of 287 requisitions (22.2%) and 1004 requisitions (77.8%) were assigned to RI-RADS A and non-A grades, respectively (Table 3). The multivariable logistic regression analysis suggested that the requisitions from the fever clinic, and thoracic surgery were less likely to be assigned to RI-RADS A than those from internal medicine. The requisitions from neurology were more likely to be assigned to RI-RADS A than those from internal medicine. The requisitions for abdomen, and head were less likely to be assigned to RI-RADS A than those of chest. The contrast-enhanced CT requisitions were more likely to be assigned to RI-RADS A than non-enhanced CT requisitions.

**Table 3:** Association between RI-RADS A grade and clinical variables

Variables	RI-RADS		*p* value	Univariable logistic analysis			Multivariable logistic analysis		
	Grade A (*n* = 287)	Grade non-A (*n* = 1004)		OR	95% CI	*p* value	OR	95% CI	*p* value
Age, year, mean ± standard deviation, median (interquartile range)	53.3 ± 22.3, 57.0 (37.5)	54.2 ± 20.9, 56.0 (33.0)	0.651	n. a.	n. a.	n. a.	n. a.	n. a.	n. a.
Gender, *n* (%)			0.129	n. a.	n. a.	n. a.	n. a.	n. a.	n. a.
Male	139 (48.3)	477 (47.5)							
Female	148 (51.8)	527 (52.5)							
Requesting specialty, *n* (%)			< 0.001						
Chest pain center	9 (3.1)	27 (2.7)		0.58	0.27–1.27	0.176	n. a.	n. a.	n. a.
Emergency medicine	29 (10.1)	79 (7.9)		0.64	0.40–1.03	0.067	0.86	0.49–1.51	0.602
Fever clinic	12 (4.2)	149 (14.8)		0.14	0.08–0.26	< 0.001	0.10	0.05–0.19	< 0.001
General surgery	23 (8.0)	136 (13.6)		0.29	0.18–0.48	< 0.001	0.58	0.32–1.05	0.072
Gynecology and obstetrics	3 (1.1)	12 (1.2)		0.44	0.12–1.57	0.205	n. a.	n. a.	n. a.
Internal medicine	129 (45.0)	225 (22.4)		Ref.					
Neurology	50 (17.4)	156 (15.5)		0.56	0.38–0.82	0.003	3.30	1.54–7.70	0.005
Neurosurgery	8 (2.8)	73 (7.3)		0.19	0.09–0.41	< 0.001	1.02	0.37–2.84	0.969
Orthopedics	2 (0.7)	95 (9.5)		0.04	0.01–0.15	< 0.001	0.00	0.00–Inf.	0.976
Otolaryngology	13 (4.5)	7 (0.7)		3.24	12.6–8.33	0.015	3.76	0.63 22.46	0.147
Thoracic surgery	3 (1.1)	33 (3.3)		0.16	0.05–0.53	0.003	0.11	0.03–0.38	< 0.001
Others	6 (2.1)	12 (1.2)		0.87	0.32–2.38	0.789	n. a.	n. a.	n. a.
Body region, *n* (%)			< 0.001						
Abdomen	53 (18.5)	263 (26.2)		0.47	0.33–0.67	< 0.001	0.30	0.19–0.47	< 0.001
Cardiovascular	29 (10.1)	26 (2.6)		2.57	1.46–4.54	0.001	0.27	0.07–1.13	0.072
Chest	133 (46.3)	307 (30.6)		Ref.					
Extremity	2 (0.7)	47 (4.7)		0.10	0.02–0.41	0.001	2.22 × 10^5^	0.00–Inf.	0.981
Head	34 (11.9)	244 (24.3)		0.32	0.21–0.49	< 0.001	0.06	0.03–0.15	< 0.001
Multiple body regions	24 (8.4)	86 (8.6)		0.64	0.39–1.06	0.082	n. a.	n. a.	n. a.
Neck	12 (4.2)	1 (0.1)		27.70	3.57–215.19	0.001	4.11	0.33–51.58	0.273
Spine	0 (0.0)	30 (3.0)		0.00	0.00–Inf.	0.971	n. a.	n. a.	n. a.
Requisition time, *n* (%)			0.003						
Office hours	144 (59.2)	470 (46.8)		Ref.					
Before midnight	112 (39.0)	394 (39.2)		0.93	0.70–1.23	0.601	n. a.	n. a.	n. a.
After midnight	31 (10.8)	140 (13.9)		0.72	0.47–1.11	0.140	n. a.	n. a.	n. a.
Contrast agent, *n* (%)			< 0.001						
With	36 (12.54)	34 (3.4)		4.09	2.51–6.67	< 0.001	3.39	1.03–11.13	0.044
Without	251 (87.46)	970 (96.6)		Ref.					

A total of 358 requisitions (27.7%) and 933 requisitions (72.3%) were assigned as adequate (RI-RADS A or B) and inadequate (RI-RADS C, D, or X), respectively (Table 4). The requisitions from chest pain center, fever clinic, general surgery, orthopedics, and thoracic surgery were less likely to be adequate than those from internal medicine; while those from neurology were more likely to be adequate. The requisitions for abdomen, cardiovascular, head, and multiple body regions were less like to be adequate than those of the chest. The requisitions after midnight were less likely to be adequate than requisitions during office hours. The contrast-enhanced CT requisitions were more likely to be adequate than non-enhanced CT requisitions.

**Table 4:** Association between adequate requisitions and clinical variables

Variables	RI-RADS		*p* value	Univariable logistic analysis			Multivariable logistic analysis		
	Grade A/B (*n* = 358)	Grade C/D/X (*n* = 933)		OR	95% CI	*p* value	OR	95% CI	*p* value
Age, year, mean ± standard deviation, median (interquartile range)	54.0 ± 21.1, 56.0 (33.0)	54.0 ± 21.6, 58.0 (35.8)	> 0.999	n. a.	n. a.	n. a.	n. a.	n. a.	n. a.
Gender, *n* (%)			0.419	n. a.	n. a.	n. a.	n. a.	n. a.	n. a.
Male	164 (45.8)	452 (48.4)							
Female	194 (54.2)	481 (51.6)							
Requesting specialty, *n* (%)			< 0.001						
Chest pain center	10 (2.8)	26 (2.8)		0.53	0.25–1.13	0.100	0.43	0.19–0.99	0.047
Emergency medicine	31 (8.7)	77 (8.3)		0.55	0.35–0.88	0.013	0.77	0.44–1.34	0.355
Fever clinic	43 (12.0)	118 (12.6)		0.50	0.33–0.75	< 0.001	0.32	0.21–0.49	< 0.001
General surgery	26 (7.3)	133 (14.3)		0.27	0.17–0.43	< 0.001	0.54	0.31–0.95	0.033
Gynecology and obstetrics	3 (0.8)	12 (1.3)		0.34	0.10–1.24	0.103	0.70	0.18–2.66	0.599
Internal medicine	149 (41.6)	205 (22.0)		Ref.					
Neurology	60 (16.8)	146 (15.6)		0.057	0.39–0.82	0.002	3.55	1.59–7.92	0.002
Neurosurgery	9 (2.5)	72 (7.7)		0.17	0.08–0.035	< 0.001	1.00	0.37–2.67	0.999
Orthopedics	5 (1.4)	92 (9.9)		0.07	0.03–0.19	< 0.001	0.11	0.02–0.81	0.030
Otolaryngology	13 (3.6)	7 (0.8)		2.56	1.00–6.56	0.051	3.09	0.53–18.12	0.211
Thoracic surgery	3 (0.8)	33 (3.5)		0.13	0.04–0.42	< 0.001	0.08	0.02–0.28	< 0.001
Others	6 (1.7)	12 (1.3)		0.69	0.25–1.87	0.465	1.87	0.58–5.99	0.295
Body region, *n* (%)			< 0.001						
Abdomen	60 (16.8)	256 (27.4)		0.33	0.24–0.47	< 0.001	0.27	0.06–0.42	< 0.001
Cardiovascular	29 (8.1)	26 (2.8)		1.58	0.90–2.77	0.110	0.24	0.06–0.96	0.044
Chest	182 (50.8)	258 (27.7)		Ref.					
Extremity	3 (0.8)	46 (4.9)		0.09	0.03–0.30	< 0.001	0.48	0.05–4.77	0.533
Head	44 (12.3)	234 (25.1)		0.27	0.18–0.39	< 0.001	0.06	0.03–0.14	< 0.001
Multiple body regions	26 (7.3)	84 (9.0)		0.44	0.27–0.71	< 0.001	0.31	0.16–0.58	< 0.001
Neck	12 (3.4)	1 (0.1)		17.01	2.19–131.98	0.007	3.71	0.30–45.87	0.306
Spine	2 (0.6)	28 (3.0)		0.10	0.02–0.43	0.002	0.31	0.03–2.72	0.290
Requisition time, *n* (%)			0.068						
Office hours	187 (52.2)	427 (45.8)		Ref.					
Before midnight	133 (37.2)	373 (40.0)		0.81	0.63–1.06	0.124	0.78	0.58–1.05	0.107
After midnight	38 (10.6)	133 (14.3)		0.65	0.44–0.97	0.036	0.62	0.40–0.97	0.036
Contrast agent, *n* (%)			< 0.001						
With	36 (10.1)	34 (3.6)		2.96	1.82–4.80	< 0.001	2.72	0.84–8.11	0.096
Without	322 (89.9)	899 (96.4)		Ref.					

### RI-RADS grade and clinical reasoning

**Fig. 3:**
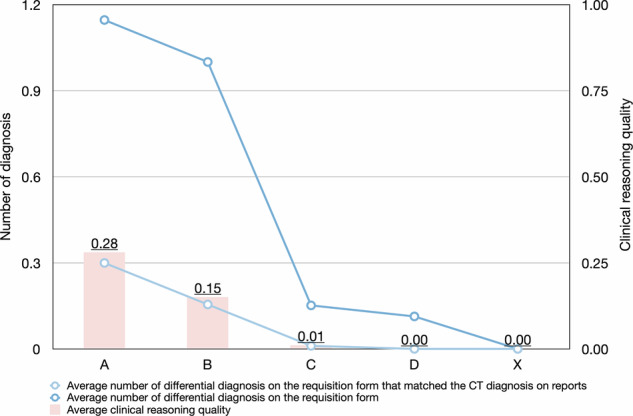
The clinical reasoning quality according to the RI-RADS grade. The number of differential diagnosis on the requisition form that matched the CT diagnosis on issued reports, and the number of differential diagnosis on the requisition form decreased with the RI-RADS grades. None of the RI-RADS D and RI-RADS X requisitions provided differential diagnosis on the requisition form. The clinical reasoning quality was higher in RI-RADS A and RI-RADS B requisitions

The average ± standard deviation, median (interquartile range) of overall clinical reasoning quality was 0.08 ± 0.26, 0.00 (0.00) (Fig. 3). The clinical reasoning quality was significantly different among RI-RADS grades (Table 5). Clinical reasoning quality was significantly higher in RI-RADS A requisitions than those of RI-RADS grades of C (adjusted *p* < 0.001), D (adjusted *p* < 0.001), and X (adjusted *p* < 0.001), but not those of RI-RADS B (adjusted *p* = 0.100). The clinical reasoning quality was better in requisitions of RI-RADS B than those of RI-RADS grades of C (adjusted *p* = 0.017), D (adjusted *p* = 0.007), and X (adjusted *p* = 0.007).

**Table 5:** The clinical reasoning quality according to RI-RADS grades

Variables	Overall (*n* = 1291)	RI-RADS					*p* value
		Grade A (*n* = 287)	Grade B (*n* = 71)	Grade C (*n* = 851)	Grade D (*n* = 53)	Grade X (*n* = 29)	
Number of differential diagnoses on the requisition form that matched the CT diagnosis on reports, mean ± standard deviation, median (interquartile range)	0.08 ± 0.28, 0.00 (0.00)	0.30 ± 0.47, 0.00 (1.00)^c,d,e^	0.15 ± 0.36, 0.00 (0.00)^c,d,e^	0.01 ± 0.10, 0.00 (0.00)^a,b,d,e^	0.00 ± 0.00, 0.00 (0.00)^a,b,c^	0.00 ± 0.00, 0.00 (0.00)^a,b,c^	< 0.001
Number of differential diagnoses on the requisition form, mean ± standard deviation, median (interquartile range)	0.41 ± 0.65, 0.00 (1.00)	1.15 ± 0.67, 1.00 (0.00)^b,c,d,e^	1.00 ± 0.17, 1.00 (0.00)^a,c,d,e^	0.15 ± 0.44, 0.00 (0.00)^a,b,e^	0.11 ± 0.32, 0.00 (0.00)^a,b,e^	0.00 ± 0.00 0.00 (0.00)^a,b,c,d^	< 0.001
The quality of clinical reasoning, mean ± standard deviation, median (interquartile range)	0.08 ± 0.26, 0.00 (0.00)	0.28 ± 0.44, 0.00 (1.00)^c,d,e^	0.15 ± 0.35, 0.00 (0.00)^c,d,e^	0.01 ± 0.10, 0.00 (0.00)^a,b,d,e^	0.00 ± 0.00 0.00 (0.00)^a,b,c^	0.00 ± 0.00 0.00 (0.00)^a,b,c^	< 0.001

^a^ Significant difference compared to Grade A

^b^ Significant difference compared to Grade B

^c^ Significant difference compared to Grade C

^d^ Significant difference compared to Grade D

^e^ Significant difference compared to Grade X

## Discussion

Our study showed the low quality of emergency CT requisitions using RI-RADS, due to incompleteness of impression, clinical information, and especially diagnostic questions. We further identified factors that are associated with a requisition with worse RI-RADS grading, including requesting specialty of fever clinic and thoracic surgery, and body region of abdomen and head. The requisitions requesting the specialty of neurology, and contrast-enhanced CT scans were associated with a better RI-RADS grading. In addition, we found that the clinical reasoning quality was better in higher RI-RADS grades than in lower ones.

RI-RADS is an effective tool for identifying inadequate requisitions with substantial agreement. Kasalak et al firstly validated the clinical usefulness of RI-RADS in a tertiary care center regardless of imaging modality [24], and found that only 23.6% of requisitions conformed to RI-RADS A grade. Parillo et al argued that only 1.0% of the inpatient diagnostic imaging referrals were RI-RADS A grade [25]. Our study demonstrated 22.2% of RI-RADS A grade in emergency CT requisitions. Although the results varied among centers, it is agreed that the majority of the radiological requisitions are of suboptimal quality. The high inter-rater agreement guarantees the clinical usage of RI-RADS for quality assurance and improvement purposes [24–26].

The potentially influencing factors on RI-RADS grade include indication for imaging, requesting specialty, imaging modality, and body region [24, 25]. Our study found that requisitions from the fever clinic and thoracic surgery were of lower quality. The clinicians of these two specialties highly rely on the CT scans to reach the diagnosis. The clinicians from the fever clinic and thoracic surgery usually request a chest CT to exclude pneumonia or rib fracture, but only provide a requisition of fever or unspecified injury without any other information. They sometimes even provide no clinical information, as they consider that radiologists should understand what diseases they are requesting to rule out. These requisitions were rated as RI-RADS with lower grades. Clinicians from the chest pain center, general surgery, or orthopedics also tend to request CT scans for chest or abdomen pain, or unspecified injury, without sufficient clinical information. Requisitions for the abdomen and head usually come without working or differential diagnoses, but ask radiologists to tell them why patients have abdominal pain or headache. Cardiovascular scans, especially CT angiography of the carotid, vertebral, and cerebral arteries, usually accompany head scans for headache. Scans covering multiple regions are often requested when clinicians are uncertain about the diagnosis. These requisitions were usually without any appropriate diagnosis and rated as RI-RADS with lower grades.

Our study confirmed that the requesting specialty and body region were associated with the RI-RADS grade, although the associated specialty and body region were not specifically the same as the previous studies. The source of variations can be attributed to the type of population and imaging modalities [24, 25]. Further, Parillo et al found that requisitions for common preoperative imaging and device check were more likely to be incomplete. However, these factors cannot be compared with the current study on emergency CT referrals. Both of the previous studies demonstrated that the indication of imaging was one of the associated factors with RI-RADS grade [24, 25]. However, our study did not investigate the influence of the indication for imaging, because most of the indications for imaging in our emergency CT requisitions were unspecified. Our study identified an extra factor of contrast usage as a factor associated with better requisition quality. We suppose that patients who are scheduled to undergo contrast-enhanced scans are more complex and severe, and are usually suggested by the radiologist after a non-contrast-enhanced scan. Therefore, clinicians may tend to provide more clinical information to reach the imaging diagnosis. Parillo et al also showed that the emergency head CT requisitions with RI-RADS grades C and D have a lower appropriateness than those with RI-RADS grades A and B, and may cause needless radiation [26]. Asking clinicians to improve their requisitions may allow them to take a second thought about CT referrals, and potentially reduce unnecessary imaging.

A potential resolution for the low-quality radiological requisitions is the assistance of large language models (LLMs). LLMs could make it possible for clinicians to enter their radiology requisitions into the electronic system and instantly receive the corresponding RI-RADS grade [29]. A locally-adapted model demonstrated high performance and almost perfect agreement with radiologists in automated RI-RADS grading [30]. This immediate feedback would allow them to modify their requisition to ensure requisition completeness and improve communication among radiologists and clinicians. LLMs have also shown the potential of extracting clinical information. The summary of clinical information generated by LLMs was more complete compared to original requisitions, and was preferred by radiologists for imaging interpretation [31, 32]. However, the written requisition forms by clinicians are still necessary as the clinical notes generated by LLMs are marginally inferior in usefulness and safety [33]. Training on how to request meaningful radiology examinations after detailed clinical reasoning is warranted to improve radiology workflow for better medical practice. Finally, it may ensure the accuracy of imaging interpretation and perceived safety.

Our study further connected radiology requisition quality and the clinical reasoning quality of clinicians. The concern of radiologists is true that low-quality radiology requisitions potentially reflect the worst clinical reasoning. In accordance with the results of the emergency abdominal CT scans [], the overall clinical reasoning quality is low. As expected, requisitions with RI-RADS A and B tend to have a higher clinical reasoning score than those with RI-RADS C, D, or X. This is related to the fact that the category of impression, which includes differential diagnoses, may be absent in the lower RI-RADS scores, whereas it should always be present in RI-RADS A and B [22, 23]. Therefore, it is reasonable that there was no significant difference in clinical reasoning scores between the RI-RADS A and B requisitions. The requisitions are recommended to have these contents to assist radiologists in achieving accurate imaging diagnosis. High-quality requisitions with differential diagnoses generally have a higher likelihood of matching the imaging diagnosis, as most of the requisitions are without any differential diagnoses that are considered by clinicians. It is necessary to inform clinicians that the clinical information in their requisitions is of great significance in the radiological diagnosis [10–16] and should be completed with thoughtful clinical reasoning. The healthcare system should take action to improve the requisition quality, including routine feedback to clinicians on their requisition quality, training courses on how to request appropriate and meaningful radiology examinations after detailed clinical reasoning [19, 34], as well as the introduction of assistance of modern tools like LLMs [29–33].

Our study has several limitations. First, it was a single-center retrospective study using data from last year. The results may not fully generalize to other settings with different volumes, types, or specialties. Further, our study focused on the emergency CT requisitions. Therefore, it may be challenging to directly compare our results of quality assessment to other clinical settings. Second, not all the clinical information was included in the requisition forms. It is also possible for radiologists to communicate with the patients or referring clinicians through phone calls, or to extract the relevant clinical history from the digital medical records. However, it may not always be possible in emergency CT requisitions because of unconscious patients, the workload of clinicians, and/or incomplete medical records. It should be noted that radiological requisitions are not only expected to be informative enough by radiologists, but also supposed to contain all necessary information to avoid the risk of errors. Third, one radiologist evaluated all the requisitions in our study. It may be more clinically practical for the requisition quality to be evaluated by the first junior reader of the radiological examination. Our inter-rater agreement indicated higher absolute agreement than the previous study [25], which also supports the assessment of requisition quality using RI-RADS by a sole radiologist. Nevertheless, the relatively low agreement in the category of clinical information needs to be modified to enable more robust evaluation. Fourth, we did not evaluate all the potential variables influencing requisition quality, such as patient expectations, severity of patients’ clinical presentations, experience of referring clinicians, and defensive medicine practices. These factors may be investigated in future studies with a prospective design. The influence of insufficient clinical information on CT reports was neither evaluated in terms of the subsequent effect on patient management nor outcome. It has been demonstrated that CT reports frequently change the diagnoses and decisions of clinicians in emergency department settings [35]. Fifth, our study only evaluated the quality of emergency CT requisitions, but not their appropriateness. Clinicians should also pay attention to requesting appropriate radiological examinations specific to the patient’s clinical history or condition. It not only reduces population exposure levels to ionizing radiation, but also optimizes healthcare resources [36, 37]. Moreover, clinical outcomes, such as patient management, and hospital stay, were not assessed. This limits the clinical relevance of the findings. These factors should be investigated in future prospective studies. Finally, the tool for assessment of clinical reasoning quality only regraded the matching of CT diagnosis and differential diagnosis on the requisition form. There is no validation or reliability data provided for this scoring method. It may not fully reflect the clinical reasoning process of clinicians. However, it is still a timely tool for clinical reasoning quality assessment, as the tool has identified a culture change in clinicians spending less effort and have become less skilled at clinical reasoning; in essence, relying more on CT scans to replace their own probabilistic hypothetico-deductive reasoning [26, 27].

In conclusion, our study found that the majority of emergency CT requisitions were inadequate, and potentially reflected worse clinical reasoning quality. Quality was lower in requisitions from the fever clinic and thoracic surgery, and those of the abdomen and head. The use of standardized requisition frameworks, like RI-RADS, may help bridge the gap between diagnostic imaging and clinical reasoning, promoting safer and more effective patient care.

## Data Availability

The datasets used and/or analyzed during the current study are available from the corresponding author on reasonable request.

## Abbreviations

LLM: Large language model
RI-RADS: Reason for exam imaging reporting and data system
